# The future of CMR: All-in-one vs. real-time CMR (Part 2)

**DOI:** 10.1016/j.jocmr.2024.100998

**Published:** 2024-01-17

**Authors:** Francisco Contijoch, Volker Rasche, Nicole Seiberlich, Dana C. Peters

**Affiliations:** a University of California, San Diego, CA, USA; b Ulm University Medical Center, Department of Internal Medicine II, Ulm, Germany; c Michigan Institute for Imaging Technology and Translation, Department of Radiology, University of Michigan, Ann Arbor, MI, USA; d Yale University, New Haven, CT, USA

**Keywords:** Cardiac MRI, Rapid imaging, Real-time imaging, Quantitative imaging, Magnetic Resonance Fingerprinting, Multitasking, Parallel imaging, Compressed sensing

## Abstract

Cardiac Magnetic Resonance (CMR) protocols can be lengthy and complex, which has driven the research community to develop new technologies to make these protocols more efficient and patient-friendly. Two different approaches to improving CMR have been proposed, specifically “all-in-one” CMR, where several contrasts and/or motion states are acquired simultaneously, and “real-time” CMR, in which the examination is accelerated to avoid the need for breathholding and/or cardiac gating. The goal of this two-part manuscript is to describe these two different types of emerging rapid CMR protocols. To this end, the vision of all-in-one and real-time imaging are described, along with techniques which have been devised and tested along the pathway of clinical implementation. The pros and cons of the different methods are presented, and the remaining open needs of each are detailed. Part 1 tackles the “All-in-One” approaches, and Part 2 focuses on the “Real-Time” approaches along with an overall summary of these emerging methods.

## Introduction

In recent years, several different schools of thought have emerged regarding reducing the length and improving accessibility of cardiovascular magnetic resonance (CMR) exams. One basic approach is to tailor the protocol specifically for a narrow clinical question, reducing the number of individual sequences that are used. As an example, a well-constructed 30-minute CMR exam [1], [2] has been developed which can address a significant number of clinical indications. While this approach is used in many institutions, it could be even further streamlined by incorporating advanced MRI methods. In Part I of this debate, we described how “All-in-One” approaches might further streamline the CMR exam, exploiting shared MRI data to generate a rich set of contrasts without increasing scan time. Here we describe the an alternative, “real-time” approach, which has long been a goal of CMR researchers. In a “Real-Time” approach, an individual sequence is collected fast enough to remove the need for breath-holding and ECG-gating, thereby making the acquisition more robust (less prone to gating errors), more comfortable for patients, and typically faster. Real-time scanning may also serve to make protocol much more efficient since it could not only accelerate individual acquisitions but also serve to eliminate delays between breathholds. The real-time approach has yet to be fully deployed clinically due to associated technical challenges, but significant progress has been made towards key portions of a fully real-time protocol.

The goal of this paper is to describe the “Real-Time” approach, and to describe its strengths and weaknesses and its current clinical utility and remaining unmet needs.

### Vision of a real-time CMR protocol

Real-time CMR aims to make CMR examinations simpler for patients, more time efficient, and more robust to the presence of arrhythmias by exploiting the tremendous progress made in accelerated MRI. This enables scanning which foregoes the conventional ECG-gated, breath-held approach and instead, relies on single-shot imaging. By developing these methods, real-time CMR also enables the possibility for new scan paradigms such as imaging during exercise, pharmacological agents (e.g. dobutamine stress [3] to unmask ischemia), or respiratory maneuvers, or other physiologic challenges. In this section, we outline each of these benefits and a vision for future efforts.

Eliminating the need for breathholds significantly improves the patient experience. It can also lead to improved image quality as it decreases the need for strict patient compliance to breathhold instructions, important e.g. for pediatric patients. The use of repeated breath-holding can be problematic for patients, especially those with advanced cardiovascular disease. In one study, 9% of patients sent to cardiac imaging could not hold their breath [4]. Free-breathing approaches can improve assessment of morphology and function (cine imaging) as well as late gadolinium enhancement (LGE) and other tissue characterization assessments in such patients. Performing free breathing scans can also improve the efficiency of scanning as it reduces the amount of table time used for patients to recover after each breathhold.

Real-time, single-shot CMR methods do not rely on a stable cardiac rhythm. As a result, real-time CMR has already frequently been used in the clinic for accurate evaluation of cardiac morphology and function in patients with arrhythmias. Not only do real-time methods enable visualization of aperiodic events such as arrhythmic beats, but they can also provide quantitative evaluation of morphology and function and avoid the need for repeat scanning due to non-diagnostic imaging when conventional gated scans are performed in patients with irregular cardiac rhythms.

While it might not yet be possible to generate an entire CMR protocol for all clinical indications [5] from real-time acquisitions, it is definitely possible to use fully real-time methods to quickly answer a single critical question, i.e. does the patient have myocardial scar, what are the ventricular or atrial volumes and EFs, what is the myocardial T2*? In these cases, a very short scan time could be employed, which would make cardiac MR more accessible, by greatly increasing throughput. In real-time protocols not reliant on ECG triggering, speed and workflow may be further improved, since ECG lead placement and use are further time-consuming complexities.

The major vision of a real-time approach is to obtain high quality diagnostic function, flow and LGE without need for reliable breath-holding or ECG-gating. The real-time protocol addresses these challenges, already today providing high quality cine, LGE, and even flow, for patients who would otherwise have non-diagnostic scans. However, not all methods have been easily transformed into real-time acquisitions. Furthermore, some single shot scans still require an ECG to begin the acquisition based on a trigger (beginning of systole). An *ECG-free* protocol where *all* needed sequences were available is another future goal of the real-time protocol. To interpret the real-time data, a stronger grasp on free-breathing “reference” values and reproducibility is needed [6]. Furthermore, in multi-slice acquisitions such as LGE and cine, the process for registration of slices acquired in different respiratory-phases has not been fully determined. Therefore, the vision of evaluation of physiology in real-time has only been partly realized, e.g., stress perfusion, function, and LGE but much more can be accomplished with real-time CMR in the future.

Real-time imaging can be used to assess for intentional changes in physiology during exercise [7], [8], respiratory-maneuvers [9], [10] or pharmaceuticals[11], for which real-time cine and flow monitoring are key. For example, real-time strain imaging with a strain-encoded (SENC) acquisition can be used to identify ischemia [12] using a stress/rest protocol. The response of ventricular septal curvature to respiration can indicate pathologic ventricular coupling, as found in constrictive pericarditis [13].

Pediatric imaging also benefits from real-time approaches[14], [15], since children are less willing to follow breath-holding instructions, or even stay still and often need to be imaged while sedated. This is all the more important, since pediatric imaging is a major CMR indication. This is somewhat more challenging due to their high respiratory and cardiac rates, compensated for partly by a smaller field of view [16]. Lastly, real-time imaging can be used for guidance of interventional procedures and could enable CMR to be used during both diagnostic and therapeutic interventions [17].

Finally, advanced approaches for real-time imaging have been used [18] to achieve another important requirement for real-time CMR: methods that do not trade-off real-time imaging capability for large losses in temporal and spatial resolution, or image quality.

### Existing methods for real-time CMR

Many different approaches have been proposed to enable real-time CMR and its application to cardiac imaging. Considering the demands of reasonable temporal and spatial resolution for a particular application, it is nearly impossible to meet the Nyquist sampling theorem with real-time. The challenge is thus to “make more from less”, which for real-time CMR translates into an efficient interplay between sampling strategies to rapidly collect as much k-space data as possible with low levels of coherent aliasing artifacts, and dedicated techniques to enable artefact-free image reconstruction from incomplete data.

The first studies of RT-CMR focused on echo-planar imaging (EPI) techniques [20], in which multiple k-space lines are acquired after a single excitation, but k-space is still encoded on a Cartesian sampling grid. Although this approach improved over the years [21], [22], [23], [24], the unfavorable point-spread-function (PSF) and inefficient use of gradients motivated the exploration of alternative k-space encoding schemes including spiral[21], [25], [26], [27] and radial[28], [29], [30], [31], [32], [33], [34] trajectories, which have favorable properties regarding residual motion during data acquisition and aliasing [35]. While for spiral imaging, a variable sampling density can be used [36], [37], major developments for radial acquisitions include the use of golden angle[38] angular increments ensuring homogeneous k-space coverage independent of the number of spokes used for reconstruction or tiny golden angle [39] to simultaneously ensure homogeneous coverage but reduce artifacts from rapid gradient switching.

**Fig. 1:**
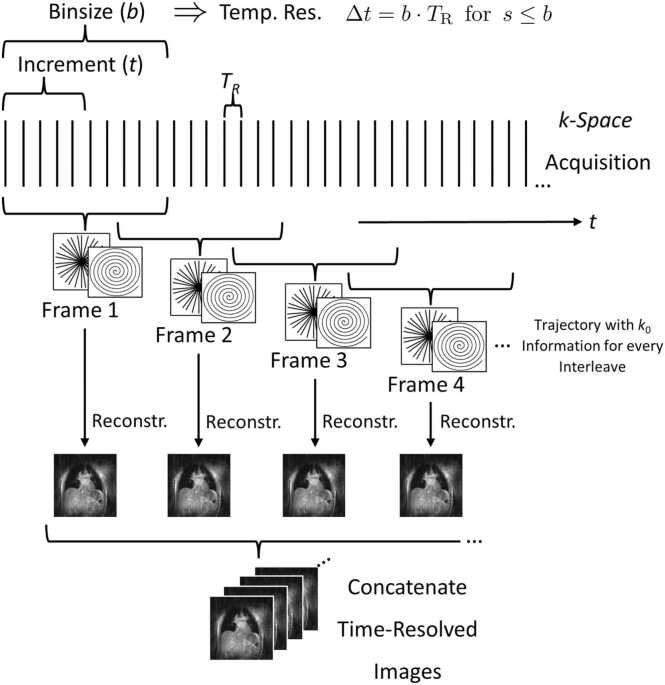
For real-time CMR, often trajectories are applied which allow image reconstruction from an arbitrary bin size,b. Prominent examples are radial and spiral trajectories, often combined with tiny or regular golden angle angular increment. For improving the “apparent” temporal resolution, the increment, t, between subsequent reconstructions is normally chosen smaller than b, yielding a classical sliding window reconstruction with almost free choice of a compromise between spatial and temporal resolution, and aliasing artifact level. CMR: cardiovascular magnetic resonance

Most real-time methods rely on these undersampled trajectories (Fig. 1) with advantageous aliasing artifacts, in combination with dedicated reconstruction techniques. Here parallel imaging [40], [41], [42], [43] and k-t BLAST with parallel imaging have been suggested and investigated in a few clinical studies[44], [45], [46], [47], [48], [49], [50], [51], [52]. These techniques enable higher degrees of undersampling either by temporal and/or spatial regularization, usage of coil sensitivity maps, or combinations thereof. In this context, sampling schemes with low-discrepant, noise-like aliasing artifacts support the successful application of the advanced image reconstruction techniques. Parallel imaging (PI) techniques, such as SENSitivity Encoding (SENSE) and GeneRalized Autocalibrating Partial Parallel Acquisition (GRAPPA), rely on complementary data provided from multiple-element coils but require additional measurements of the coil-sensitivity maps (for SENSE) or the reconstruction weights (for GRAPPA). These techniques also can be paired with non-Cartesian trajectories to enable higher acceleration factors [53], [54]. The need for additional calibration data can be avoided by using temporal SENSE/GRAPPA approaches, which derive the respective information from a sliding window reconstruction [53], [55], or by deploying self-calibration approaches [56]. K-t techniques use spatiotemporal redundancy of the data, where analysis of the frequency components (f) of the temporal evolution of a certain spatial position (x) in so-called x-f space can assist in the removal of respective aliasing artifacts before the data are transformed back into the k-t representation. PCA analysis has been introduced for constraining the reconstruction [57] and the problem has also been addressed by modelling the MR signal as separable into spatial and temporal dimension and formulation of the reconstruction as low-rank matrix recovery problem [58], [59]. For further acceleration, k-t approaches such as UNaliasing by Fourier-encoding the Overlaps in the temporaL Dimension (UNFOLD) [60] have been combined with parallel imaging[61], [62]. High acceleration factors of up to 4 (PI) and even 8 (k-t) have been reported. However, the need for calibration scans and/or residual temporal blur have led to the development of techniques simultaneously estimate the coil sensitivities and image content either by alternative minimization using Joint image reconstruction and SENsitivity Estimation (JSENSE) [63] or a more general regularized non-linear inverse (NLINV)[64] problem.

Compressed sensing (CS) [65] has also been proposed as an acceleration approach independent of parallel imaging-based approaches for real-time CMR. CS utilizes a non-linear reconstruction technique assuming a sparse representation in a specific transformation of the object in spatial or temporal domain in combination with incoherent sampling. By collecting data along trajectories such as golden angle radial sampling acceleration factors of up to 13 have been reported in combination with PI and CS reconstructions [39].

Future directions in real-time CMR most-likely include the application of deep learning (DL) based approaches. In CMR, DL approaches have recently yielded unprecedented undersampling rates. Even though a rather new field, the substantial body of literature ([66], [67] and references therein) clearly indicate the potential of DL approaches for outperforming the conventional PI and CS techniques, in reconstruction speed, and possibly quality, by either aiming at the direct reconstruction of k-space data (end-to-end) or artifact removal in the reconstructed images. The major advantage of DL techniques results from the general concept of using application-tailored data-driven spatiotemporal priors which efficiently support the reconstruction task for cardiovascular application. Most DL approaches learn through supervised learning and require a training set of fully sampled artifact-free data. In this approach, it is ideal to have a set of fully-sampled datasets that will be used as ground truth for training the network. Even though of high potential, DL-based reconstruction is facing specific challenges for real-time CMR, mainly caused by the dynamic nature of the data (respiratory and cardiac), which increases the complexity as well as limits the availability of suitable training data [68].

### Evidence for the value of real-time CMR: Validation and Deployment of real-time methods

#### Real-time cine

Real-time CMR has been proven clinically feasible with a strong focus on left-ventricular function assessment. The main clinical utility of real-time cine imaging is in severely arrhythmic or non-cooperative patients. Over the past twenty years, real-time cine has become a reality in the clinic, providing similar masses, volumes and EFs, with strong correlations and Bland-Altman limits of agreement for patients, compared to breath-hold cine (**see** Table 1 **showing patient studies**). Adults and children, and subjects with and without arrhythmia have been studied. Agreement has been strong, at the level of interobserver agreement for breath-hold bSSFP cine itself, which typically has a low bias near 0, and low variance ( ± 2SDs = ± 16 mls) for volumes [72] in adults.

**Table 1:** Performance of real-time cine in chronological order shows progress.

Authors,Year, Subjects	Approach Details	Results, LOA	Cardiac and respiratory
**Adults**			
Hori et al. 2003[114]. 18 patients and 6 controls.	Low spatiotemporal resolution bSSFP with 63 lines Resolution: 4x3mm^2^ and 91 ms,	**Bias± 2SDs** LVEDV:3 ± 18mls LVESV: 8 ± 18mls LVEF: − 4 ± 9% LVM:− 9 ± 11 g	ECG-triggered, free-breathing
Aandal et al.[50]. 2014 63 patients	Radial GRAPPA, 2.3 x 2.2 mm^2^, 43.8 ms	**Bias± 2SDs** LVEDV:− 2.6 ± −26.4mls LVESV: − 0.3 ± −16.7mls LVEF: − 0.9 ± 4.4%	**ECG-free, free-breathing**
Contijoch et al.[116] 2015, 18 clinical patients	Golden angle radial bSSFP, 1.7 – 2.3 x 1.7 – 2.3x 8 mm^3^, ∼50 ms (variable).	**Bias± 2SDs** LVEDV: 5.2 ± 24mls LVESV: 0.8 ml± 11mls LVSV: 4.3 ± 25mls LVEF: 0.6 ± 10%	**ECG-free, free-breathing, end-expiratory data selected.**
2016 Kido et al. 81 patients.	Cartesian CS with bSSFP vs. conventional, both 1.7 x1.7 x6mm^3^ and 41 ms. reconstruction with Har wavelets.	**Bias± 2SDs** LVEDV:− 1 ± 15mls LVESV: 0.2 ± 10mls LVSV:1.1 ± 11mls LVEF − 4 ± 5% LVM: 1 ± 12 g	**ECG-triggered, breath-held.**
Sudarski et al. 2016 60 patients 58 years± 14. 42/60 male, 9 with arrythmias. [76]	Single-shot bSSFP accelerated using CS at 3 T, compared to reference.2x2x8mm^3^, 34 ms	**Bias± 2SDs** LVEDV:1.9 ± 19.2mls LVESV: 0.8 ± 22mls LVSV:2.3 ± 19.6mls LVEF: 1 ± 7.2% LVM: − 8.7 ± 21 g Similar for non BH real-time cine.	**ECG-triggered, both breath-held and non- breath-held.**
Haji-Valizadeh et al. 2018 16 patients with MI, 18 patients with chronic kidney disease, 15/34 male. Age ∼60 years old. (1.5& 3 T)	Radial k-t SPARSE 1.5 T, tiny golden angle, with 32 ms temporal resolution and 2x2x8mm^3^ for RT, vs. 35 ms temporal resolution and 2x2x6mm^3^ for reference breath-held cardiac cine	**Bias± 2SDs** For patients at 1.5 T LVEDV: 15 ± 30mls LVESV: 1 ± 2 25 mls LVSV:3 ± 24 mls LVEF: − 3 ± 9% LVM: 7 ± 25 g Similar for non BH real-time cine.	**ECG-triggered** **free-breathing**
Nita et al.[45] 2022 32 patients, 11 with arrythmia, 57 ± 16 year old, 59% male.	Spiral with golden angle reconstructed with K-t SPARSE SENSE. RV also studied. Compared to Echo measurements. 2x2x8mm^3^, and 40 ms.	LVEDV: 1 ± 7.4mls LVESV: 0.1 ± 8.8 mls LVSV:0.2 ± 9 mls LVEF: 1 ± 5.5% LVM: 7 ± 25 g	ECG-triggered (?), Free-breathing
Laubrock et al. 2022[46] 59 controls and AF patients at 3 T. AF 30 patients at 1.5 T, age 69 + /− 9 (patients) years, 34% female.	Radial bSSFP NLIINV vs. conventional bSSFP. 1.3-1.5 x 1.3-1.5mx 6-8 mm^3^, 40 ms temporal resolution (33 ms for 3 T RT).	**Bias± 2SDs** LVEDV:− 8 ± 25mls LVESV: − 6 ± 21mls LVSV:− 1.2 ± 26mls LVEF 2 ± 17% LVM: 12 ± 33 g Better for controls. Better image quality for real-time in patients with arrythmia.	**ECG-free, free-breathing**
**Pediatric studies**			
Steeden et al.[80] 2018 60 children 14 ± 3 years old, 45% male	Spiral bSSFP with tiny golden angle with CS reconstruction. 1.7x 1.7 mm^2^ and 30 ms temporal resolution.	**Bias± 2SDs** LVEDV:2 ± 8 mls LVESV: − 1 ± 7mls LVSV: 3 ± 9.5mls LVEF 2 ± 6	**ECG-triggered, free-breathing**
Zucker et al. 2021[15] 50 children, 16 ± 4 years old, 60% M.[50]	bSSFP with optimized acquisition order reconstructed with deep learning. Also studied RV volumes.	**Bias± 2SDs** LVEDV:1 ± 23mls LVESV:− 1 ± 16mls LVSV: 2 ± 18mls LVEF: 1 ± 10%	**Free-breathing respiratory-triggered.**
Rower et al.[50] 2022 22 pediatric patients at 1.5 T. 13 ± 4 years old, 36% male.[15]	2D bSSFP conventional vs. real-time with binning. 1.6x1.6x8mm, 33 ms temporal resolution. RV also studied	**Bias± 2SDs (Indexed values)** LVEDVi: 0.1 ml/m^2^± 2.7 ml/m^2^ LV-ESVi: 0.4 ml/m^2^± 1.9 ml/m^2^ LVSVi: 0.5 ml/m^2^± 2.3 LVEF: − 0.5% ± 1.6% Mean BSA∼ 1.6 m^2^	**ECG and respiratory (bellows) monitoring for post-hoc binning**
Hatipoglu et al.[79] 2022 55 pediatric patients, average 12 years old, 62% male. [80]	Breath-held single shot bSSFP cine with CS vs. conventional, no late diastolic evaluation. 1.5x 1.9 mm^2^, 40 ms.	**Bias± 2SDs** LVEDV: 3.6 ± 9.5 mls LVESV: 1.3 ± 7mls LVEF: 0.1% ± 5%	**ECG-triggered, breath-held.**
[79]			

*bSSFP* balanced steady state free precession, *CS* compressed sensing, *GRAPPA* generalized autocalibrating partially parallel acquisitions, *SENSE* sensitivity encoding.

Even very early studies with low temporal resolution showed such excellent correlations [73] of LV and RV volumes and masses, approaching the “scan-rescan/inter-observer limit” noted above. This is partly because the metrics most commonly measured (and vitally important) in CMR, LV end-systolic and end-diastolic volumes and EF, are somewhat robust to temporal and spatial blur, such that computed tomography (CT), with lower temporal resolution [74], showed good agreement with MRI (bias ± 2SDs=1.2% ± 5% for EF with CT). This suggests that, for developers of real-time CMR, it might be important to identify validation metrics which are more challenging, when evaluating real-time cine, such as measuring atrial contraction, the slope of LV filling in early diastole [75], LV and LA strains and strain rates by feature tracking, or RV volumes, which were not well measured by CT in the recent report [74]. For such tasks, further improvements in temporal and spatial resolution and quality for real-time cine are needed. Table 1 shows a progression towards use of more advanced techniques and improved temporal and spatial resolution.

These findings of agreement have been repeated when performing real-time cine with parallel imaging, low-rank reconstructions, radial acquisitions and CS, and deep learning. For example, using commercially available CS for real-time cine with spatial and temporal resolution comparable to breath-held cine, good agreement of functional metrics was obtained [76]. Earlier studies using customized CS methods [77] also demonstrated similar results.

**Fig. 2:**
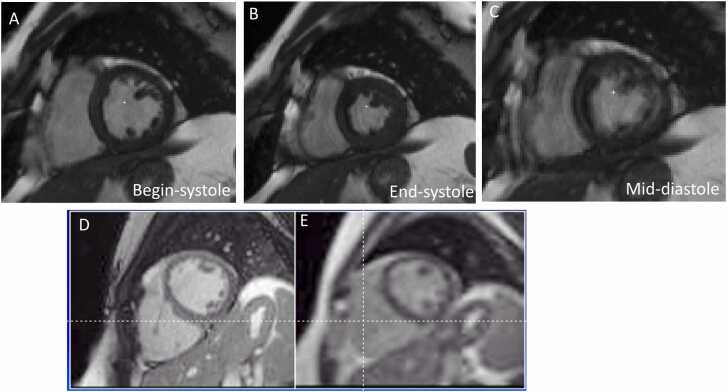
Cine in a patient (49 y.o. man with atrial fibrillation) experiencing arrhythmia during the MRI exam shows acceptable quality during systole (A-B), but strong artifacts by mid-diastole (C), using conventional retrospective ECG-gated cine, which can be avoided using prospective ECG-gating or real-time imaging. Another subject (36 y.o. man with bigeminy), where conventional cine (D) was obtained by scanning over two heart-beats with 1.6x 1.6 x 6 mm^3^ resolution, and (E) real-time cine (3x3x8mm^3^ resolution) was also acquired, providing similar information.

**Fig. 3:**
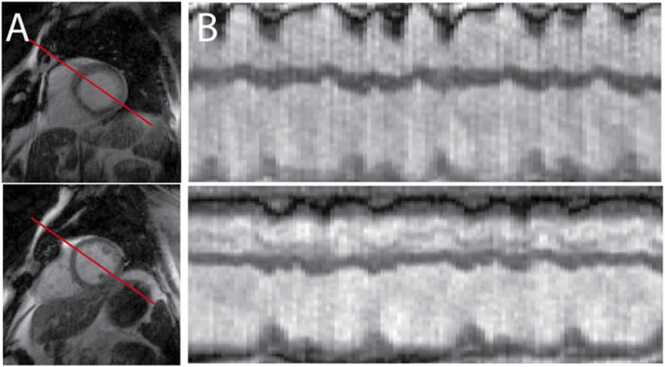
Left ventricular function of a subject with an arrhythmia as imaged using a real-time sequence. A) Two short-axis slice locations are shown with temporal projections (B) as shown by the red line. The irregular heart rhythm led to impaired segmented cine acquisition and the irregular pattern can be visualized with real-time imaging. Images taken from Contijoch et al. [116].

Fig. 2A-C shows segmented, retrospectively ECG-gated cine in an arrhythmic patient. The image quality appears unaffected in systole, but the effect of arrhythmia can easily be observed in mid-diastole, where quality is poor, because of the binning of incompatible data. Importantly, the value of real-time cine has also been validated in arrhythmic patients (such as in Fig. 2D-E). A recent study of patients with atrial fibrillation (an irregular arrhythmia) [46] found that 13% of conventional cine images were non-interpretable. In patients who had diagnostic cine, the resulting measurements of conventional breath-hold and real-time cine agreed well. Of course, for the arrhythmic patients, there was no gold standard available for comparison to real-time cine. To address the challenge that arrhythmic beats are different than sinus rhythm beats, and that there is no single end-systolic or end-diastolic volume for patients with arrythmia, real-time techniques have been developed that evaluate function in each beat separately [18]. Fig. 3 shows such real-time imaging in a patient with arrythmia.

As noted above and evident in Table 1, another challenge for real-time cine and its validation, is that multi-slice cine imaging during free-breathing will result in unregistered slices. Because of this, some real-time cine approaches resort to use of a single breath-hold for full stack of cines. Another challenge is identifying begin-systole, which can be considered the frame with the “largest LV cavity”, but that is not as precisely determined as a QRS complex, for example considering an apical slice. The importance of choosing begin and end-systole based on *global* maximal and minimal volume has been investigated [78]. For this reason, some approaches use an initial ECG-trigger to initiate the cine scan. However, this means an ECG is required for the scan, complicating the exam.

Pediatric imaging also benefits from real-time cine and several studies exist validating the approach [15], [50], [79], [80] (Table 1). Results are similar to those for intra and interobserver variability of these metrics [81] in congenital heart disease.

#### Real-time phase-contrast

**Fig. 4:**
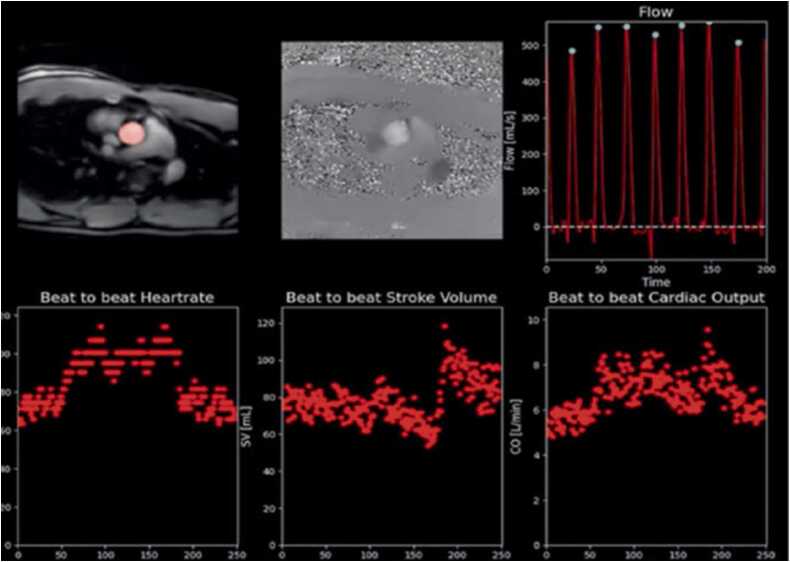
Example of a real-time monitoring method which can be used to evaluate changes in cardiac performance during a 3 min exercise protocol. The top row shows magnitude (left) and phase (middle) images with a flow curve on the right. Beat-to-beat changes in heart rate (left), stroke volume (middle), and cardiac output (right) are shown in the bottom.

Real-time phase contrast enables CMR to measure flow in patients with arrhythmia or those who cannot hold their breath[82], [83]. It also offers the opportunity to monitor response to stress, exercise and other physiologic provocations. This sequence is more challenging to acquire in real-time than cine, since the TRs are longer (e.g. 5 vs. 3 ms), and the basic acquisition is at least doubled in time, due to need for both reference and velocity encoded k-space. A very early real-time method used spiral k-space at 0.5 T [84] to measure aortic blood flow in real-time. Another such study used EPI to generate 5x5mm^2^ resolution, 40 ms temporal frames, and studied large artery and vein flow while undergoing Valsalva maneuver [85]. Many acceleration methods have since been applied to phase-contrast imaging [86], including k-t view sharing [87], parallel imaging[88], compressed sensing, low-rank reconstructions[58], and deep-learning [89], [90], with testing primarily focused on aortic flow, and demonstration of good temporal and spatial resolution. Temporal resolution is critical in phase-contrast [91], and flow wave-forms, especially flow peaks, are highly sensitive to actual temporal resolution. Real-time flow waveforms have been used to demonstrate differences in flow in consecutive beats for arrhythmic patients [92]. Just as for measurement of cardiac function, measurement of physiologic changes during flow using real-time acquisitions might be clinically valuable. For example, Fig. 4 shows real-time flow in the aorta, in which the stroke volume can be monitored, and is seen to rapidly increase during exercise.

Figure reproduced with permission from [90].

#### Real-time late gadolinium enhancement and relaxometry

**Fig. 5:**
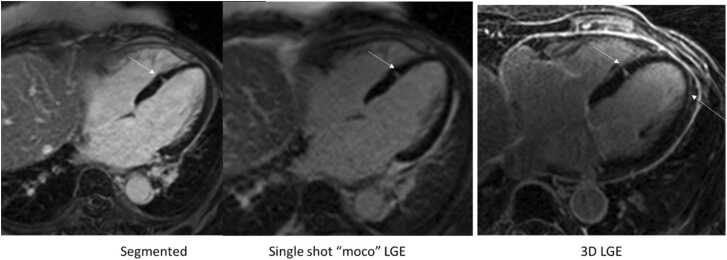
A small region of enhancement on LGE in a patient (66 year old woman with suspected myocarditis), which can be well observed on both conventional breath-hold 2D LGE (1.6 ×2.2x8mm^3^), free-breathing MoCo (1.7 ×2.2 ×8 mm^3^), and high resolution navigator-gated 3D LGE (1.5 ×1.5 ×3.6 mm^3^). However, a midwall line of enhancement on the free wall of the myocardium can only be observed with high resolution 3D LGE, highlighting the value of a high-resolution methods, still not available with real-time acquisitions.

Real-time approaches to LGE simply use free-breathing, single shot acquisitions, usually with ECG-triggering to time the acquisition to mid-diastole or systole. This real-time approach to LGE is vital in some patients who cannot hold their breath, or who have arrhythmias. Studies have shown that the infarct/scar detection and infarct size are similar using free-breathing vs. breath-held segmented approaches. Free-breathing LGE (with or without motion correction (MoCo)) is powerful, because it is robust both to poor breath-holders and possibly less sensitive to artifacts in the presence of arrhythmia [93], [94], since longer regrowth times between inversions are possible, so there is less concern regarding magnetization regrowth variability due to arrhythmia (which prevents nulling of normal myocardium and generates artifacts especially from the enhanced skin [94]). Soon after the importance of LGE was established, it was shown that free-breathing LGE was possible, using single-shot [95] or navigator-gated [96] methods. The signal-to-noise ratio (SNR) of single-shot LGE can be boosted using a bSSFP readout although the value of bSSFP vs. GRE is not definitive [97]. In a study of 200 patients [98], single-shot bSSFP LGE (without MoCo) had a strong sensitivity and specificity for infarct (88% and 95%). Clinically available free-breathing “MoCo” LGE [99], [100] uses multiple single-shot acquisitions of LGE, and registers the data from multiple heart-beats before averaging [101]. This improved quality over single-shot (single heart-beat) LGE and improved the accuracy of infarct sizing moderately [100]. A study of 390 patients compared MoCo LGE to conventional segmented breath-held LGE [101]. The spatial resolution and acquisition window for both the breath-held LGE and free-breathing LGE were similar. Results showed no bias and a small variance regarding scar volume (bias ± 2 SD= 0 ± 5 g) compared with breath-hold LGE, and shorter total scan times by a factor of two (since rest-periods between breath-holds were not needed) and better image quality scores for free-breathing. The authors noted that patients with the poorest quality breath-hold scans tended to be sicker, and therefore most in need of evaluation, highlighting the importance of free-breathing LGE. However, a limitation of that study was that MoCo LGE was consistently acquired after segmented LGE, at which time contrast is improved. Another study of 400 patients with either segmented or MoCo LGE also noted improved quality [102] and shorter scan time using MoCo LGE. Finally, a third study from a different group confirmed these findings [103]. Therefore, there is strong evidence that free-breathing LGE is accurate and effective. Fig. 5 shows that free-breathing MoCo and conventional 2D breath-held LGE both were capable of visualizing a small region of enhancement in a 67 year old female patient with suspected myocarditis. However, neither method detected a midwall enhancement that was visible with high resolution 3D LGE, highlighting a limitation of both real-time and 2D LGE.

While requirements of relaxometry make it not fully compatible with the real-time CMR approach, it is possible to use MoCo or respiratory-gating to acquire T1- [104], [105], T2- [106] and T2 * [107] during free-breathing, and more work is needed. Furthermore, ECG-gating is still used in these approaches, and relaxation values might depend on cardiac phase.

### Advantages of real-time CMR

As noted in Table 2, real-time imaging fundamentally aims to reduce the time needed for the collection of each individual frame of an image, such that imaging can be decoupled from cardiac and/or respiratory motion. Although not always the primary goal, it can also lead to shorter exams. This is already a powerful clinical tool in imaging patients with arrythmias, patients who cannot hold their breath, and children, especially for function and viability. Real-time CMR combined with a streamlined exam might improve accessibility and cost-effectiveness of CMR further. Furthermore, work is underway to develop real-time monitoring of physiological changes, which may lead to new diagnostic tests for CMR [7], [8], [9], [10], [12], [108].

**Table 2:** Advantages and disadvantages of real-time CMR.

Advantages	Disadvantages
**Immune to poor breath-holding and arrythmias.**	**Low temporal and spatial resolution—and acceleration artifacts**
Rapid scans mean there is no corruption due to breath-holding. The acquisitions are not segmented into different heart-beats, so arrythmias cannot introduce errors, artifacts and blurring.	To achieve rapid imaging, some temporal and spatial resolution are sacrificed. Furthermore, all acceleration methods introduce some artifacts or reductions in image quality.
**Rapid acquisition, and workflow improved if ECG is not need.**	**Not all CMR evaluations can be performed using real-time.**
Cine imaging can literally take 10 s. Adding setup of ECG increases complexity and set up time.	Flow and parametric mapping are possible but not thoroughly developed for real-time. ECG-gating is often still utilized for real-time protocols.
**Able to identify physiological changes.**	**Post-processing and interpretation are more challenging**
In real-time CMR, physiological variations due to exercise, breathing maneuvers, drugs, can all be evaluated in real-time.	There exist limited reference values for free-breathing scans. End-systole and end-diastole are harder to identify. Multi-slice acquisitions may have misregistered data.

*CMR* cardiovascular magnetic resonance, *ECG* electrocardiogram.

### Disadvantages of real-time CMR

In real-time CMR, **acceleration reduces** image quality, typically sacrificing some temporal and spatial resolution as shown in Table 1. Furthermore, increased artifacts are generated with rapid scanning. For example, parallel imaging can reduce scan time but the spatial location/coverage of coils may affect image quality in certain regions (through g-factor noise) or orientations. Methods such as parallel imaging, compressed sensing and machine learning reconstructions leverage prior information to reduce the amount of data that needs to be acquired. Such approaches have shown significant ability to accelerate real-time imaging. While theoretically, real-time methods could adopt an All-in-One approach [109], this has not been developed, so currently real-time methods do not leverage shared information, as is possible with All-in-One approaches. Furthermore, in general, real-time CMR relaxometry (or even free-breathing arrhythmia-insensitive mapping) is not well developed. The problem of slice-misregistration when acquiring a stack of real-time cines during free-breathing is often not discussed. A final drawback is that, especially for flow and cine, but possibly also for other contrasts, the breath-holding status changes physiology, and the volumes and EFs that are measured during breath-holding differ from free-breathing values; they might even be considered more realistic. For example, breath-holds decrease oxygenation of the blood, necessitating an increase in cardiac output, e.g. changing from 4.5 L/min (free-breathing) to 6 L/min (breath-hold) in one study [110], while a shallow breath-hold flow evaluation produces results more similar to free-breathing. There are fewer studies of “reference” values for free-breathing scans and different physiological states. For native relaxometry, the cardiac phase of acquisition might also affects values, and again there are no reference standards for this.

As described above, current CMR protocols acquire an array of sequences, each with their own parameters and technical and physiologic considerations. Accelerating these sequences may further increase the complexity of scanning (especially when trying to maximize acceleration). This may further **increase the already heavy burden placed on the technologist and CMR reader**. A myriad of sequences, each with a customized number of slices, orientations, k-space trajectories, and different resolution, creates a complex workflow that can result in errors, repeat scans or even incomplete imaging. Lastly, the technologist’s and reader’s review of images to ensure quality may become more complicated as acceleration methods are used. For example, **over-acceleration with a temporal regularization** may lead to “sluggish” cardiac motion, which is inaccurate. It may also lead to spatial smoothing which may obscure small areas of LGE or abnormal tissue mapping. Detecting these artifacts and deciding whether to repeat a scan increases the burden for CMR practitioners. Many of these disadvantages are listed in Table 2.

### Open Needs for a Fully Real-Time CMR Protocol

There are several obstacles which prevent the routine use of real-time CMR. Often, real-time methods rely on computationally expensive reconstruction techniques. This can limit on-the-fly reconstruction and respective assessment of the image quality. Real-time imaging can also result in hundreds if not thousands of images (often obtained with unknown respiratory and ECG stage) with analysis that is not currently automated. On-the-fly real-time CMR image reconstruction may be approached by the application of dedicated graphics processing unit (GPU)-based reconstructors [111], or the use of deep-learning techniques [112], [113], [117], both successfully tested in few clinical studies, but still lacking full integration into the vendors clinical systems. For more efficient analysis of the data, synchronization with the ECG [45] and/or respiration [50] is mandatory for selecting data from the same cardiac and respiratory phase for functional quantification with available analysis packages. Even though in principle possible, integration in the vendors reconstruction and analysis pipelines is often still missing.

Even if the integrational aspects are addressed, the reliability and full automatization of the real-time approaches may remain a major limiting challenge. Even though a huge variety of real-time CMR techniques have been reported in research, a standardized approach for data acquisition and analysis is still missing and needs further investigation before broad clinical application. For example, most reconstruction approaches introduced so far demand careful choice of weighting for the regularization terms, which currently clearly limits the maximal achievable acceleration factors, still yielding reliable and fully automatic image reconstruction. Once these open needs are addressed on the clinical systems, real-time CMR will likely be even more often used as an attractive alternative to conventional breath-held and gated techniques, offering improved patient comfort and the capability for accurate assessment of the heart even in arrhythmic or non-cooperative patients.

## Conclusion

CMR is a powerful tool for the evaluation of the heart, and new technologies including “all-in-one” and real-time acquisitions may further increase the efficacy of CMR and access to this imaging modality. Real-time techniques, in which each individual sequence is accelerated to avoid the need for ECG-gating and breath-holding, have been deployed and assessed for a variety of patients. Real-time cine and phase-contrast imaging, as well as free-breathing LGE imaging, are currently in use for uncooperative patients or those suffering from arrhythmias. However, these methods have yet to be incorporated into routine clinical protocols as a first choice, and so whether they can streamline an already compact CMR protocol is not clear. Furthermore, the utility of real-time protocols for monitoring physiological changes is still mostly unexplored. As an intermediate step, a move from traditional imaging to real-time imaging for some acquisitions (cine, phase-contrast, LGE) and “some-in-one” for others (T1 mapping, T2 mapping, fat fraction mapping) may provide clinicians the confidence required to make a further change to a more extensively accelerated protocol. These technologies currently exist in a research setting and are now appropriately mature for a critical clinical assessment. Through the deployment and automation of these emerging techniques, CMR may move from a complex examination only available at high-end institutions to an accessible test available for more and sicker cardiac patients.

## Ethics approval and consent to participate

Not applicable.

## Consent for publication

Not applicable.

## Funding

NIH 1R01HL144706, Development of MR-derived parameters of LV diastolic function: Validation and Comparison to LV and LA fibrosis. NIH 1R01HL162671, Improved MRI guidance of pediatric catheterization via autonomous multi-beat data synthesis. Authors' contributions: FC, VR, DP and NS were substantially involved in the conception, research, and writing of the manuscript. All authors read and approved the final manuscript.

## CRediT authorship contribution statement

**Peters Dana C.:** Conceptualization, Writing – original draft, Writing – review & editing. **Rasche Volker:** Conceptualization, Writing – original draft, Writing – review & editing. **Contijoch Francisco:** Conceptualization, Writing – original draft, Writing – review & editing. **Seiberlich Nicole:** Conceptualization, Writing – original draft, Writing – review & editing.

## Declaration of Competing Interest

The authors declare that they have no known competing financial interests or personal relationships that could have appeared to influence the work reported in this paper.

## Data Availability

Not applicable.
